# Plasma‐Induced Oxygen Defect Engineering in Perovskite Oxide for Boosting Oxygen Evolution Reaction

**DOI:** 10.1002/smll.202404239

**Published:** 2024-09-02

**Authors:** Kaiteng Wang, Jun Zhou, Lei Fu, Yunqing Kang, Zilin Zhou, Yonghong Cheng, Kai Wu, Yusuke Yamauchi

**Affiliations:** ^1^ Center of Nanomaterials for Renewable Energy State Key Laboratory of Electrical Insulation and Power Equipment Xi'an Jiaotong University Xi'an 710049 P. R. China; ^2^ Research Center for Materials Nanoarchitectonics (WPI‐MANA) National Institute for Materials Science 1‐1 Namiki Tsukuba Ibaraki 305‐0044 Japan; ^3^ Department of Materials Process Engineering Graduate School of Engineering Nagoya University Nagoya 464‐8603 Japan; ^4^ Nanozyme Laboratory in Zhongyuan Henan Academy of Innovations in Medical Science Zhengzhou Henan 451163 P. R. China; ^5^ Department of Chemical and Biomolecular Engineering Yonsei University 50 Yonsei‐ro, Seodaemun‐gu Seoul 03722 South Korea; ^6^ Australian Institute for Bioengineering and Nanotechnology (AIBN) The University of Queensland Brisbane Queensland 4072 Australia

**Keywords:** oxygen evolution reaction, oxygen vacancy, perovskite oxide, plasma

## Abstract

Perovskite oxides are considered highly promising candidates for oxygen evolution reaction (OER) catalysts due to their low cost and adaptable electronic structure. However, modulating the electronic structure of catalysts without altering their nanomorphology is crucial for understanding the structure‐property relationship. In this study, a simple plasma bombardment strategy is developed to optimize the catalytic activity of perovskite oxides. Experimental characterization of plasma‐treated LaCo_0.9_Fe_0.1_O_3_ (P‐LCFO) reveals abundant oxygen vacancies, which expose numerous active sites. Additionally, X‐ray photoelectron spectroscopy and X‐ray absorption fine structure analyses indicate a low Co valence state in P‐LCFO, likely due to the presence of these oxygen vacancies, which contributes to an optimized electronic structure that enhances OER performance. Consequently, P‐LCFO exhibits significantly improved OER catalytic activity, with a low overpotential of 294 mV at a current density of 10 mA cm^−2^, outperforming commercial RuO_2_. This work underscores the benefits of plasma engineering for studying structure‐property relationships and developing highly active perovskite oxide catalysts for water splitting.

## Graphical abstract

Plasma etching is an efficient technique for surface modification of catalysts. N_2_ plasma‐treated LaCo_0.9_Fe_0.1_O_3_ (P‐LCFO) reveals abundant oxygen vacancies and exposes many active sites, leading to excellent catalytic performance in the anodic oxygen evolution reaction of water electrolysis. This provides a simple and effective strategy for developing highly active perovskite oxide catalysts with defect engineering for water electrolysis.

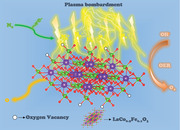

## 1 Introduction

With the continuous growth of the new energy industry, traditional fossil fuels are gradually being replaced by wind, nuclear, hydrogen, and tidal energy.^[^ ^1^, ^2^ ^]^ Hydrogen has garnered widespread attention due to its high mass‐energy density (142 MJ kg^−1^) and minimal environmental impact. Green hydrogen is primarily produced through water electrolysis. However, the efficiency of electrolytic hydrogen production is significantly hindered by the sluggish four‐electron transfer process at the anode, known as the oxygen evolution reaction (OER).^[^ ^3^, ^4^, ^5^ ^]^ Traditional OER catalysts are predominantly noble metal oxides such as RuO_2_ and IrO_2_.^[^ ^6^, ^7^ ^]^ While these materials offer good catalytic performance, noble metals are scarce and expensive, highlighting the urgent need for more cost‐effective and highly active OER catalysts.^[^ ^8^, ^9^, ^10^, ^11^ ^]^

Among the various catalysts, perovskite oxides are considered the most promising candidates to replace noble metal oxides for OER due to their flexible electronic structure and abundant components.^[^ ^12^, ^13^, ^14^, ^15^ ^]^ However, their activity is still insufficient.^[^ ^16^ ^]^ Several strategies have been proposed to enhance OER performance, including heteroatom doping,^[^ ^17^, ^18^, ^19^, ^20^ ^]^ oxygen defects engineering,^[^ ^21^, ^22^, ^23^, ^24^, ^25^ ^]^ and surface reconstruction.^[^ ^26^, ^27^, ^28^, ^29^ ^]^ Among them, oxygen vacancy engineering has been widely reported for its ability to effectively regulate the electronic structure of perovskite oxides, balance the adsorption and desorption capabilities of B‐site metal atoms for active intermediates, and alter electron mobility.^[^ ^30^, ^31^ ^]^ For example, Jiaqi Ran *et al.* reported that incorporating fluorine atoms into LaCoO₃ through calcination at 400 °C for 2 h not only introduces a certain amount of oxygen vacancies, thereby exposing more active sites, but also alters the spin state of Co^3+^, both of which contribute to improving the electrocatalytic performance of LaCoO_3_.^[^ ^32^ ^]^ Similarly, Min Lu *et al.* demonstrated that the content of oxygen vacancies in the catalyst can be regulated by controlling the milling time of perovskite oxides (La_x_Sr_1‐x_CoO_3‐δ_). This adjustment allows for the selective switching between the adsorbate evolution mechanism and the lattice oxygen oxidation mechanism.^[^ ^33^ ^]^ However, these methods of introducing oxygen vacancies typically involve high‐temperature calcination and mechanical stress treatment, which are not only time‐consuming but also inevitably lead to structural collapse and particle aggregation.

Plasma is a multi‐component active species generated at ambient or low pressure,^[^ ^34^ ^]^ which can efficiently modify nanomaterials without altering their nanostructures. For instance, He Li *et al.* reported an increase in oxygen vacancies on the surface of NiCo_2_O_4_ nanowires coated on carbon paper after H_2_/Ar plasma treatment. The treated catalyst exhibited higher conductivity and improved intrinsic activity, thereby enhancing the electrocatalytic performance of NiCo_2_O_4_ nanowires for OER and ORR.^[^ ^35^ ^]^ The advantages of plasma engineering make it applicable in a wide range of fields, including energy conversion, optoelectronics, and nanoparticle exsolution.^[^ ^36^, ^37^, ^38^ ^]^ This provides a suitable platform for studying the structure‐property relationship of catalysts.^[^ ^39^ ^]^ However, there is still a lack of studies on plasma‐induced catalytic OER activities in perovskite oxides.

**Figure 1:**
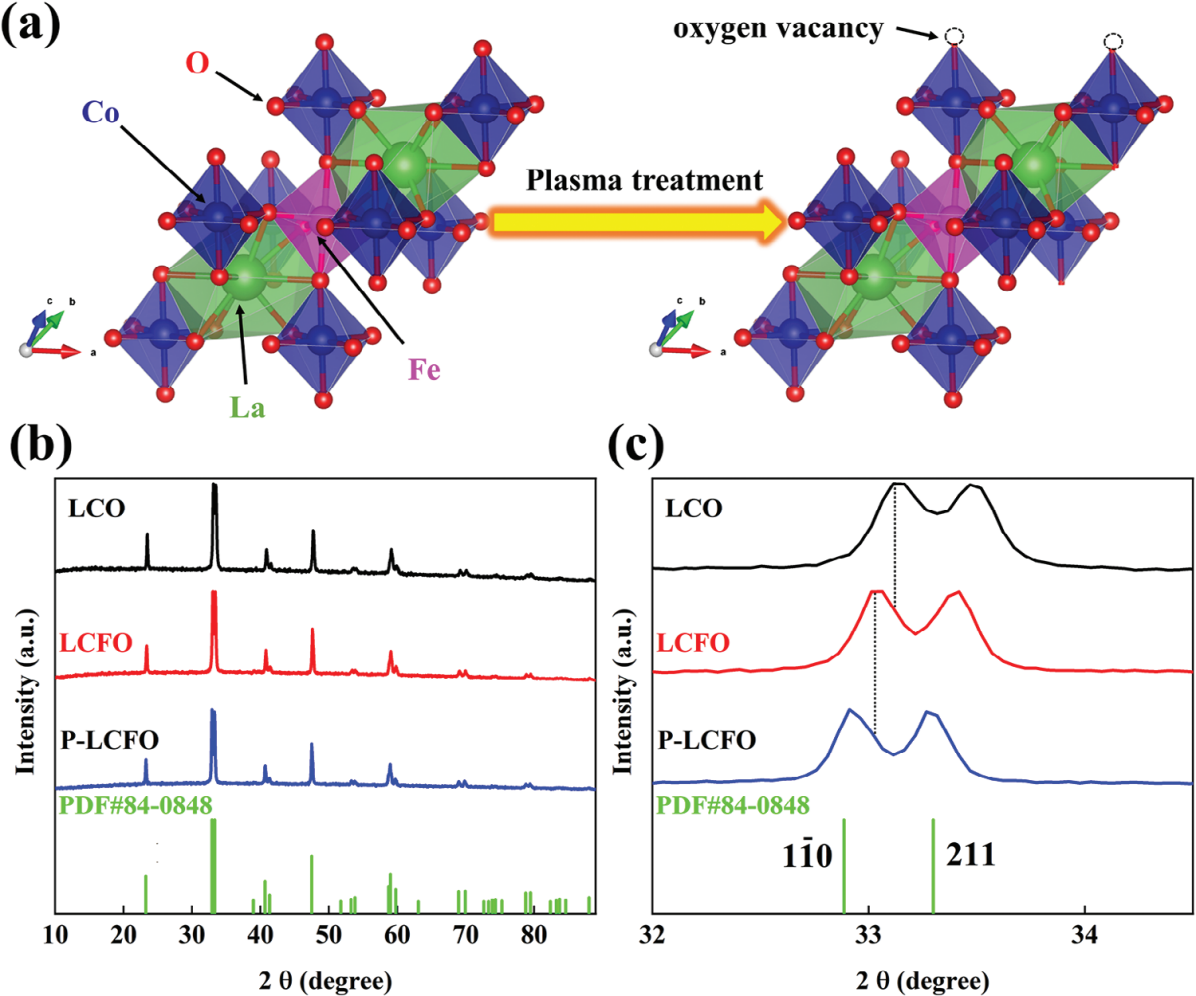
a) Schematic of the plasma treatment process. b) XRD patterns of LCO, LCFO, and P‐LCFO. c) Comparison of diffraction angles of LCO, LCFO, and P‐LCFO at 32 to 34°.

Herein, we report the synthesis of an oxygen vacancy‐rich perovskite oxide, plasma‐treated LaCo_0.9_Fe_0.1_O_3_ (P‐LCFO), using a straightforward plasma irradiation method (Figure S1, Supporting Information). Experimental characterization reveals abundant defect structures and oxygen vacancies in P‐LCFO after plasma bombardment (**Figure** **1**a), leading to the exposure of numerous active sites. Furthermore, spectroscopic analysis shows that the reduced Co oxidation state optimizes the electronic structure, enhancing OER performance. As a result, the P‐LCFO exhibits improved OER catalytic activity, achieving a low overpotential of 294 mV at a current density of 10 mA cm^−2^, which surpasses that of commercial RuO_2_. This work offers a simple yet effective strategy for developing highly active perovskite oxide catalysts through defect engineering for water electrolysis.

## 2 Results and Discussion

### 2.1 Characterization of Morphology and Electronic Structure

The X‐ray Diffraction (XRD) patterns of LaCoO_3_ (LCO), LaCo_0.9_Fe_0.1_O_3_ (LCFO), and P‐LCFO are shown in Figure 1b,c. These diffraction peaks can be indexed to an orthorhombic crystal structure (PDF#84‐0848).^[^ ^40^ ^]^ Compared to intrinsic LCO, the main peak of LCFO shifts negatively, indicating lattice expansion. This expansion is likely due to the larger ionic radius of Fe^3+^ compared to Co^3+^, further confirming Fe incorporation.^[^ ^41^ ^]^ After plasma treatment of LCFO, the diffraction peak of the (1$\bar{1}$0) crystal plane^[^ ^42^ ^]^ shifts further negatively, suggesting further lattice expansion. This may be attributed to the increase in oxygen vacancies, which simultaneously causes the reduction of Co^3^⁺ to Co^2^⁺ with a larger ionic radius to maintain electroneutrality.^[^ ^43^ ^]^ Additionally, a decrease in peak intensity for P‐LCFO indicates lower crystallinity (Figure S2, Supporting Information). The low crystallinity corresponds to a flexible long‐range disorder and a short‐range ordered structure, exposing numerous active sites.^[^ ^44^ ^]^ Moreover, the presence of defect structures enhances the catalyst's affinity for oxygen, facilitating the continuous adsorption and dissociation of intermediates during OER.^[^ ^45^ ^]^

**Figure 2:**
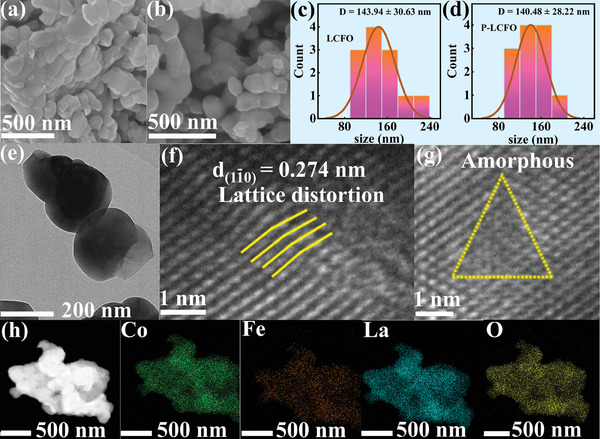
a,b) SEM images of LCFO (a) and P‐LCFO (b). c,d) Particle size distribution images of LCFO (c) and P‐LCFO (d). e–g) TEM image (e) and HRTEM images (f) and (g) of P‐LCFO. h) Elemental mapping images of P‐LCFO.

Scanning electron microscopy (SEM) images show that both LCFO and P‐LCFO are composed of irregular particles and grains (**Figure** **2**a,b). However, particle size analysis from the SEM images reveals that the grains of P‐LCFO are smaller (Figure 2c,d), suggesting a higher specific surface area and more active sites after plasma treatment. The corresponding transmission electron microscope (TEM) images are shown in Figure S3 (Supporting Information), and the sizes of individual particles in the TEM images are consistent with those observed in the SEM images (Figure 2e; Figure S4, Supporting Information). High‐resolution TEM (HRTEM) images show that the lattice fringe of P‐LCFO (0.274 nm) on (1$\bar{1}$0) crystal plane is larger than that of LCFO (0.271 nm)^[^ ^42^ ^]^ (Figure 2f; Figure S5, Supporting Information), which is consistent with the XRD results. Notably, a small amount of lattice distortion and an amorphous area appear in P‐LCFO after plasma treatment (Figure 2f,g). Elemental mapping images indicate that La, Co, Fe, and O elements are uniformly distributed throughout both LCFO and P‐LCFO (Figure 2h; Figures S5 and S6, Supporting Information), and plasma treatment did not cause any elemental segregation in the catalyst. Additionally, the N_2_ adsorption and desorption curves of LCFO and P‐LCFO are shown in Figure S7 (Supporting Information). The obtained isotherms show nitrogen uptake at higher relative pressures, indicating macropore structures for both LCFO and P‐LCFO. The Brunauer‐Emmett‐Teller (BET) specific surface area measurements of LCFO and P‐LCFO are 2.66 and 2.85 m^2^ g^−1^, respectively, indicating a slight increase in specific surface area after plasma treatment.

**Figure 3:**
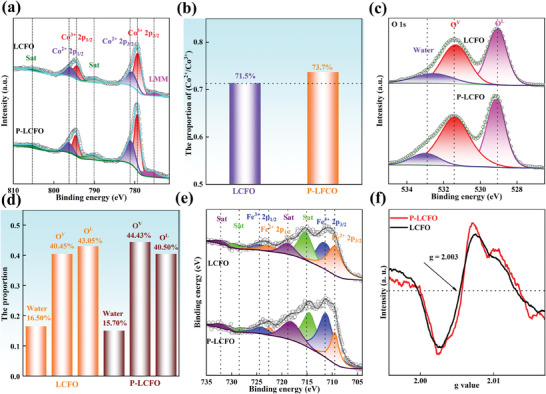
a) Co *2p* XPS spectra of LCFO and P‐LCFO. b) The proportion of (Co^2+^ /Co^3+^) of LCFO and P‐LCFO. c) O *1s* XPS spectra of LCFO and P‐LCFO. d) The proportion of different forms of oxygen of LCFO and P‐LCFO. e) Fe *2p* XPS spectra of LCFO and P‐LCFO. f) EPR spectra of LCFO and P‐LCFO.

To analyze the electronic states of the elements on the sample's surface, X‐ray photoelectron spectroscopy (XPS) spectra were collected, as presented in Figure S8 (Supporting Information). As shown in **Figure** **3**a, the peaks at 775.3, 779.4/794.4, 781.2/796.5, and 790.1/805.4 eV correspond to LMM Auger peak of Co, Co^3+^, Co^2+,^ and satellite peaks, respectively. Notably, P‐LCFO shows a higher content of Co^2+^ compared to LCFO, likely due to the increase in oxygen vacancies, which lowers the Co valence state to maintain electroneutrality (Figure 3b).^[^ ^43^ ^]^ In Figure 3c, the peaks at 529.1, 531.4, and 532.7 eV correspond to lattice oxygen (O^L^), oxygen vacancy (O^V^), and adsorbed water, respectively. Figure 3d shows a significant increase in oxygen vacancies in P‐LCFO after plasma treatment, leading to the exposure of abundant active sites and consequently enhancing catalytic activity.^[^ ^45^ ^]^ In Fe 2*p* spectra, the peaks at 709.5/722.9 eV, 711.5/724.2 eV, 714.9/728.4 eV, and 718.9/732.3 correspond to Fe^2+^, Fe^3+^ and satellite peaks (Figure 3e). As shown in Figure S9 (Supporting Information), P‐LCFO has a higher content of Fe^3+^ compared to LCFO, which supports the maintenance of the lower Co valance state. The increase in Fe valence state contributes to the synergistic effect between Co and Fe, enhancing OER performance.^[^ ^46^, ^47^, ^48^ ^]^ Additionally, electron paramagnetic resonance (EPR) was used to further investigate defect changes after plasma treatment (Figure 3f). A clear signal at g = 2.003 suggests the presence of oxygen vacancies.^[^ ^49^, ^50^ ^]^ The increased intensity observed in P‐LCFO indicates a higher concentration of oxygen vacancies after plasma treatment, which is favorable for enhancing catalytic activity.

**Figure 4:**
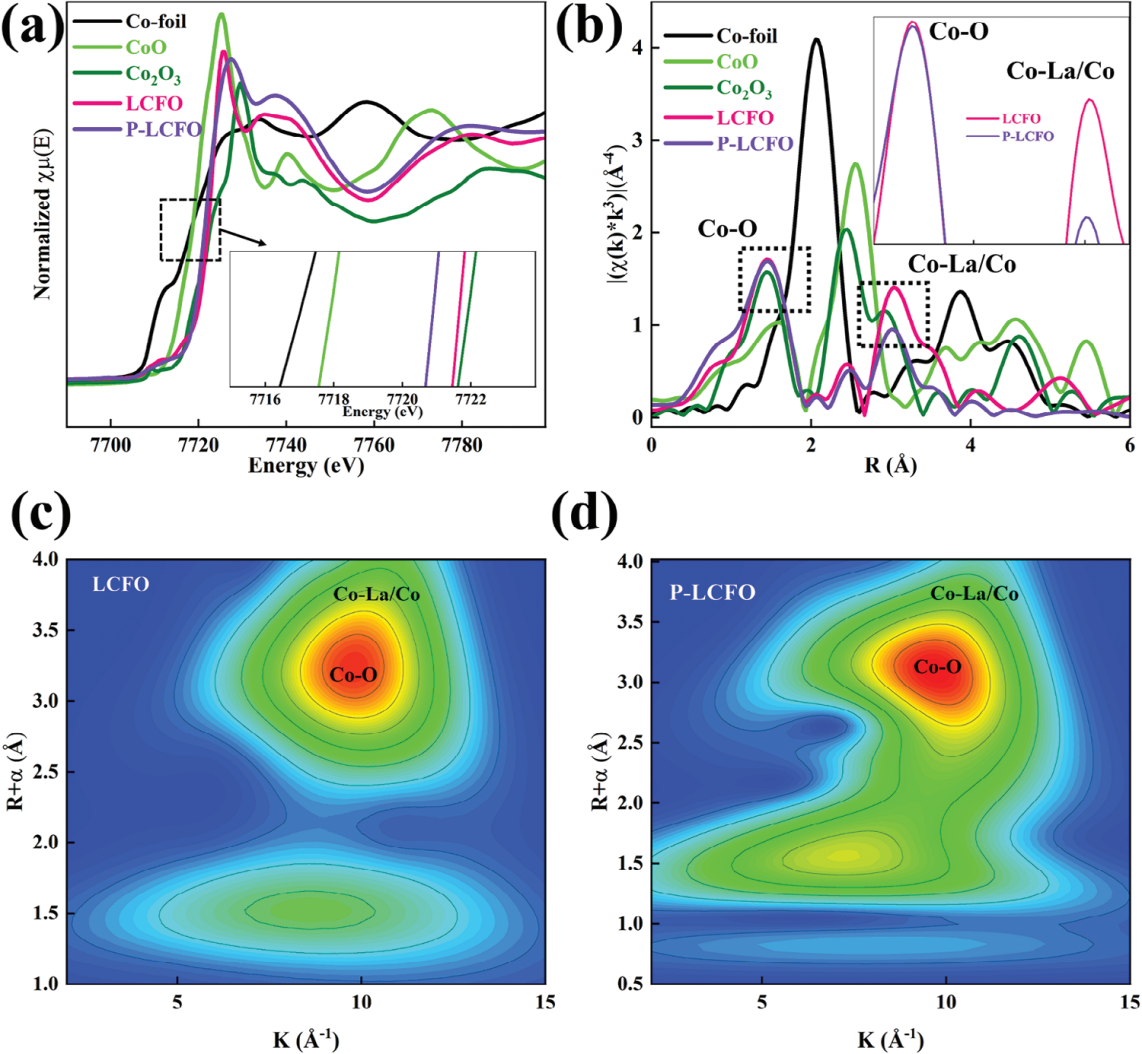
a) XANES spectra of Co K edges in LCFO and P‐LCFO. b) EXAFS spectra of Co K edges in LCFO and P‐LCFO. c,d) The high‐resolution wavelet‐transform counterplots of LCFO and P‐LCFO.

X‐ray absorption fine structure (XAFS) analysis was conducted to further understand the electronic structure of Co  in LCFO and P‐LCFO. As shown in **Figure** **4**a, the Co K‐edge X‐ray absorption near edge structure (XANES) spectrum of P‐LCFO is shifted to a lower energy region compared to LCFO, indicating that the average valence state of Co in P‐LCFO is slightly lower than that in LCFO, consistent with the XPS results.^[^ ^46^ ^]^ The extended X‐ray absorption fine structure (EXAFS) of Co K‐edge is presented in Figure 4b. The scattering signals of the Co−O and Co‐La/Co coordination shells are located at 1.4 and 3.0 Å, respectively, and these signals become weaker in P‐LCFO. As shown in Figure S10 and Table S1 (Supporting Information), the EXAFS fitting curve reveals that the bond length of Co─O and Co‐La/Co increases, and the atomic coordination number decreases in P‐LCFO, suggesting an increase in oxygen vacancies.^[^ ^51^ ^]^ These results indicate that oxygen vacancies in LCFO increase after plasma treatment, weakening the interaction between Co and O.^[^ ^52^ ^]^ High‐resolution wavelet‐transform contour plots are provided in Figure 4c,d, further verifying the electronic structure differences between LCFO and P‐LCFO.

### 2.2 Electrocatalytic Properties

**Figure 5:**
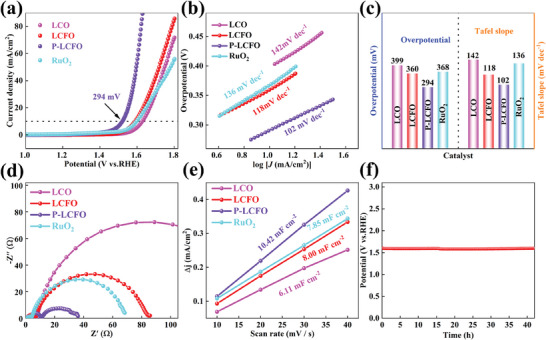
a) LSV curves, b) Tafel slopes curves, and c) comparison of overpotentials and Tafel slopes of LCO, LCFO, P‐LCFO, and the commercial RuO_2_ catalyst. d) EIS and e) C_dl_ of the LCO, LCFO, P‐LCFO, and the commercial RuO_2_ catalyst. f) Chronopotentiometry test of P‐LCFO at 10 mA cm^−2^.

The OER catalytic activity was investigated in an O_2_‐saturated 1 m KOH solution, with the reversible hydrogen electrode (RHE) serving as the reference for all electrode potentials. Commercial RuO_2_ was chosen as the reference OER catalyst for comparison with the perovskite oxide catalysts. The overpotentials of LCO and LCFO are 399 and 360 mV, respectively (**Figure** **5**a), suggesting that Fe inclusion may enhance OER activity. After plasma treatment for 30 min, P‐LCFO exhibited the lowest overpotential of 294 mV, outperforming commercial RuO_2_ (368 mV). This indicates that the increase in oxygen vacancies after plasma treatment significantly enhances the OER process. Additionally, P‐LCFO demonstrates comparable OER activity in alkaline solution to some recently reported perovskite oxides (Figure S11 and Table S2, Supporting Information).^[^ ^53^, ^54^ ^]^ To evaluate the kinetic of the OER, the Tafel slope was analyzed (Figure 5b,c). P‐LCFO shows a lower Tafel slope (102 mV dec^−1^) compared to LCO (142 mV dec^−1^), LCFO (118 mV dec^−1^), and commercial RuO_2_ (136 mV dec^−1^), suggesting improved kinetics for P‐LCFO. The Nyquist diagram of the catalyst is shown in Figure 5d. An equivalent circuit for EIS fitting indicates (Figure S12 and Table S3, Supporting Information) that P‐LCFO has the smallest charge transfer resistance (R_ct_) among all tested samples, further supporting that the electron transfer process is accelerated after plasma treatment, thereby enhancing catalytic performance. As shown in Figure S13 (Supporting Information), the double layer capacitance (C_dl_), used to predict the electrochemical active surface area (ECSA), was obtained through cyclic voltammetry conducted at various scan rates. Figure 5e indicates that the C_dl_ of P‐LCFO is 10.42 mF cm^−2^, larger than its counterparts, due to the exposure of more active sites on the P‐LCFO surface after plasma treatment. Additionally, the Faraday efficiency of P‐LCFO is ≈94.6% at a current of 50 mA (Figure S14, Supporting Information). The chronopotentiometry curve (Figure 5f) shows a slight potential change, confirming good stability over 42 h.^[^ ^55^ ^]^ EDS mapping and HRTEM images of P‐LCFO after the stability test reveal that La, Co, Fe, and O elements are still uniformly distributed throughout P‐LCFO, with some defect structures still present (Figures S15 and S16, Supporting Information). The O *1s* XPS spectrum of P‐LCFO shows no significant change in oxygen vacancy content after stability testing (Figure S17, Supporting Information). This stability could be attributed to the amorphous surface of the catalyst after plasma treatment, which accelerates surface oxygen exchange and lattice oxygen migration, thereby enhancing OER stability.^[^ ^56^ ^]^ Consequently, the excellent catalytic activity and stability of P‐LCFO make it a promising candidate for the development of efficient and cost‐effective OER catalysts.

## 3 Conclusion

In summary, we have reported a facile method for introducing defects on the surface of perovskite oxides using plasma technology. N_2_ plasma can be employed to bombard the surface of LCFO, increasing the concentration of oxygen vacancies. Experimental characterization reveals abundant defect structures in P‐LCFO after plasma treatment. XAFS and XPS results indicate that a low Co valence state, along with a significant number of oxygen vacancies, optimizes the electronic structure, contributing to the improvement of OER performance. Consequently, P‐LCFO exhibits enhanced OER activity, achieving a low overpotential of 294 mV at a current density of 10 mA cm^−2^. This work underscores the benefits of plasma engineering for exploring structure‐property relationships and developing highly active perovskite oxide catalysts for water splitting.

## Conflict of Interest

The authors declare no conflict of interest.

## Supporting information

Supporting Information

## Data Availability

The data that support the findings of this study are available from the corresponding author upon reasonable request.
