# Supplementary material for: Plasma‐Induced Oxygen Defect Engineering in Perovskite Oxide for Boosting Oxygen Evolution Reaction
Source: Small. 2024 Sep 2;20(48):2404239. doi: 10.1002/smll.202404239 (PMC11600700; doi:10.1002/smll.202404239)
Supplement: Supplementary file 1 — Supporting Information [file SMLL-20-2404239-s001.docx]

Supporting Information

**Plasma-Induced Oxygen Defect Engineering in Perovskite Oxide for Boosting Oxygen Evolution Reaction**

Kaiteng Wang, ^a^ Jun Zhou, ^a, *^ Lei Fu, ^a, b, c *^ Yunqing Kang, ^c, d^ Zilin Zhou, ^a^ Yonghong Cheng, ^a^ Kai Wu, ^a^ Yusuke Yamauchi, ^c, e, f *^

*a Center of Nanomaterials for Renewable Energy, State Key Laboratory of Electrical Insulation and Power Equipment, Xi’an Jiaotong University, Xi’an 710049, People’s Republic of China*

*b* *Research Center for Materials Nanoarchitectonics (WPI-MANA), National Institute for Materials Science, 1-1 Namiki, Tsukuba, Ibaraki 305-0044, Japan*

*c Department of Materials Process Engineering, Graduate School of Engineering, Nagoya University, Nagoya 464-8603, Japan*

*d Nanozyme Laboratory in Zhongyuan, Henan Academy of Innovations in Medical Science, Zhengzhou* *451163, Henan, China*

*e Department of Chemical and Biomolecular Engineering, Yonsei University, 50 Yonsei-ro, Seodaemun-gu, Seoul 03722, South Korea*

*f Australian Institute for Bioengineering and Nanotechnology (AIBN), The University of Queensland, Brisbane, Australia*

**Experimental section**

**Materials**

Lanthanum nitrate (La(NO_3_)_3_·6H_2_O), cobalt nitrate (Co(NO_3_)_2_·6H_2_O), ferric nitrate (Fe(NO_3_)_3_·9H_2_O), and potassium hydroxide (KOH) were procured from Aladdin. Nafion solution (Nafion D520 (5%)) was procured from Suzhou Sinero Technology Co., Ltd. All the chemicals were used straightly without further purification.

**Catalysts synthesis**

LaCoO_3_ (LCO) and LaCo_0.9_Fe_0.1_O_3_ were prepared by sol-gel method. 20 mmol La(NO_3_)_3_·6H_2_O, 18 mmol Co(NO_3_)_2_·6H_2_O, and 2 mmol Fe(NO_3_)_3_·9H_2_O were weighed and added to 200 ml DI water solution with a total metal ions-to-citric acid ratio of 3: 1. After stirring and evaporating at 180 °C for 5h, it was transferred into an oven at 200 °C for foaming for 5 h. The foamed sample was sintered in an 800 °C muffle furnace for 6 h to obtain the precursor perovskite oxide.

The precursor perovskite oxide was placed in the dielectric barrier discharge DBD plasma treatment instrument (**Figure S1**), and the N_2_ gas was introduced for about 30 minutes. The surface modification can be completed at 160 V voltage and 200 W power for 30 minutes.

**Characterization and** **treatment of the catalysts**

The crystal phase was analyzed by Bruker D8 Advance X-ray diffractometer (XRD) and the angle of 2**θ** from 0° to 90°. The morphology of catalysts was characterized by SEM and TEM using Zeiss Gemini500 field emission scanning electron microscope and FEI Talos F200x transmission electron microscope. EPR tested by Bruker EMXplus-6/1 paramagnetic resonance spectrometer. The electronic states were examined by XPS using a Thermo Fisher ESCALAB Xi^+^. The X-ray absorption spectra (XAS) including X-ray absorption near-edge structure (XANES) and extended X-ray absorption fine structure (EXAFS) of the samples were collected at the Singapore Synchrotron Light Source (SSLS) center, where a pair of channel-cut Si (111) crystals was used in the monochromator. The storage ring was working at the energy of 2.5 GeV with an average electron current of below 200 mA The plasma experimental equipment is CTP-2000KP, and the high-voltage DC power supply is DW-P503-1ACDF.

**Electrochemical measurement**

All electrochemical measurements were performed using a classic three-electrode system, with an electrochemical workstation (CHI660E) in 1 M KOH solution. The graphite rod and Hg / HgO electrode were selected as the counter electrode and the reference electrode, respectively, and the glassy carbon electrode (GC, 5 mm) was used as the working electrode. The preparation method of the working electrode for the catalytic reaction is as follows: 4 mg of catalyst and 1.5 mg of carbon black (XC-72) were dispersed in a mixed solution of DI water (10 μL), ethanol (470 μL) and 5 wt% Nafion solution (20 μL) through sonication for 45 min. Then 20 μL of the ink was dropped on the glassy carbon electrode. Linear sweep voltammetry (LSV) was performed at 10 mV s^−1^, with an 85% iR correction. The electrode potential was standardized to a reversible hydrogen electrode ( RHE, E_RHE_ = E_Hg / HgO_ + 0.098 + 0.059 pH ). The electrochemical active surface area (ECSA) is positively correlated with the double-layer capacitance (C_dl_), and the potential is selected in the range of 0.1 ~ 0.2 V (vs. Hg / HgO) in the non-faradaic process. CV curves were obtained at continuous scanning rates of 10, 20, 30, and 40 mV s^−1^. The electrochemical impedance spectroscopy(EIS) test was performed in a frequency range of 100 kHz to 0.01 Hz with an amplitude of 5 mV. Carbon paper was used as the working electrode(catalyst loading of 0.64 mg cm^−2^ ) for stability test.


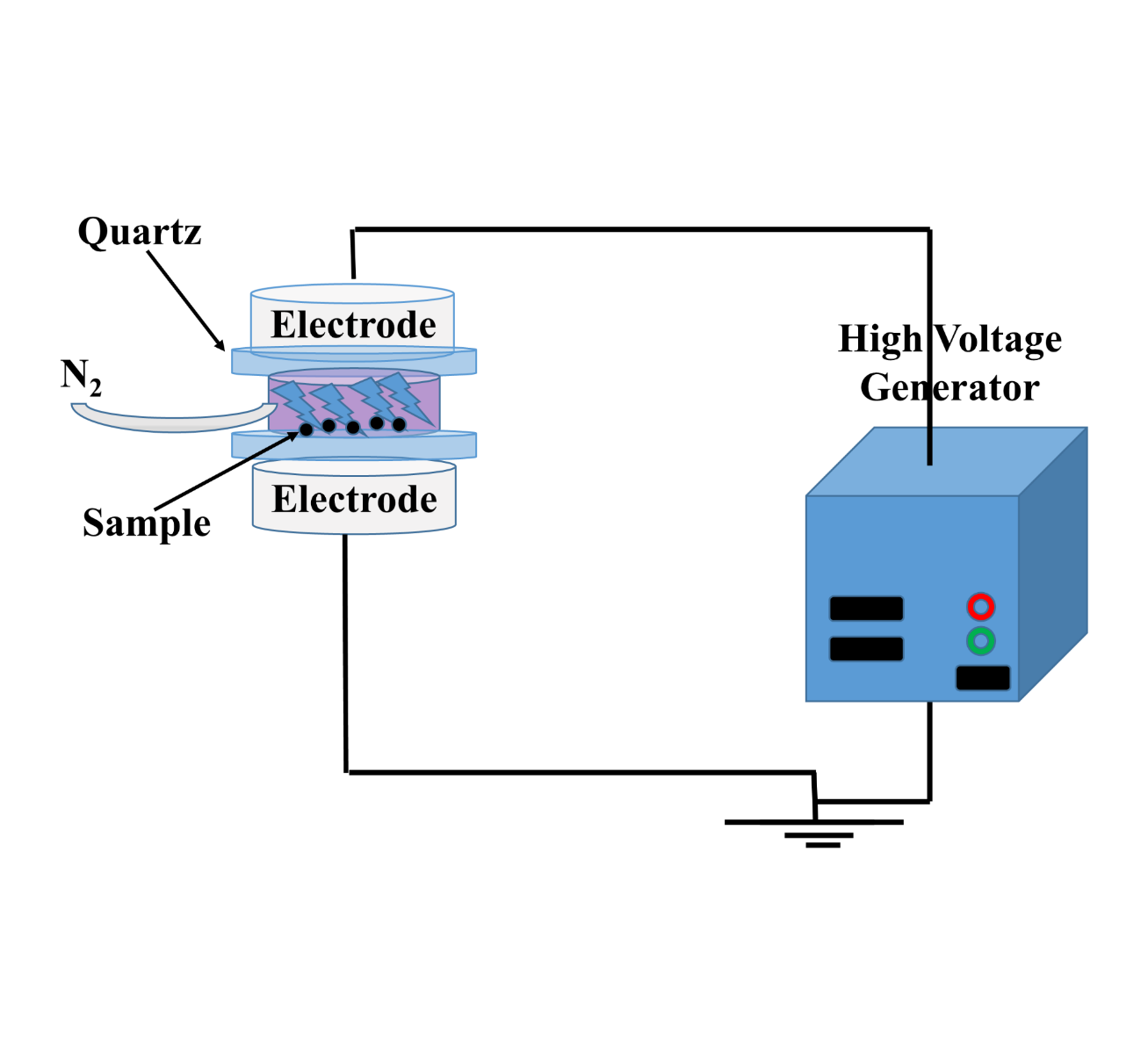


**Figure S1.** Schematic of Dielectric Barrier Discharge (DBD) plasma treatment device.


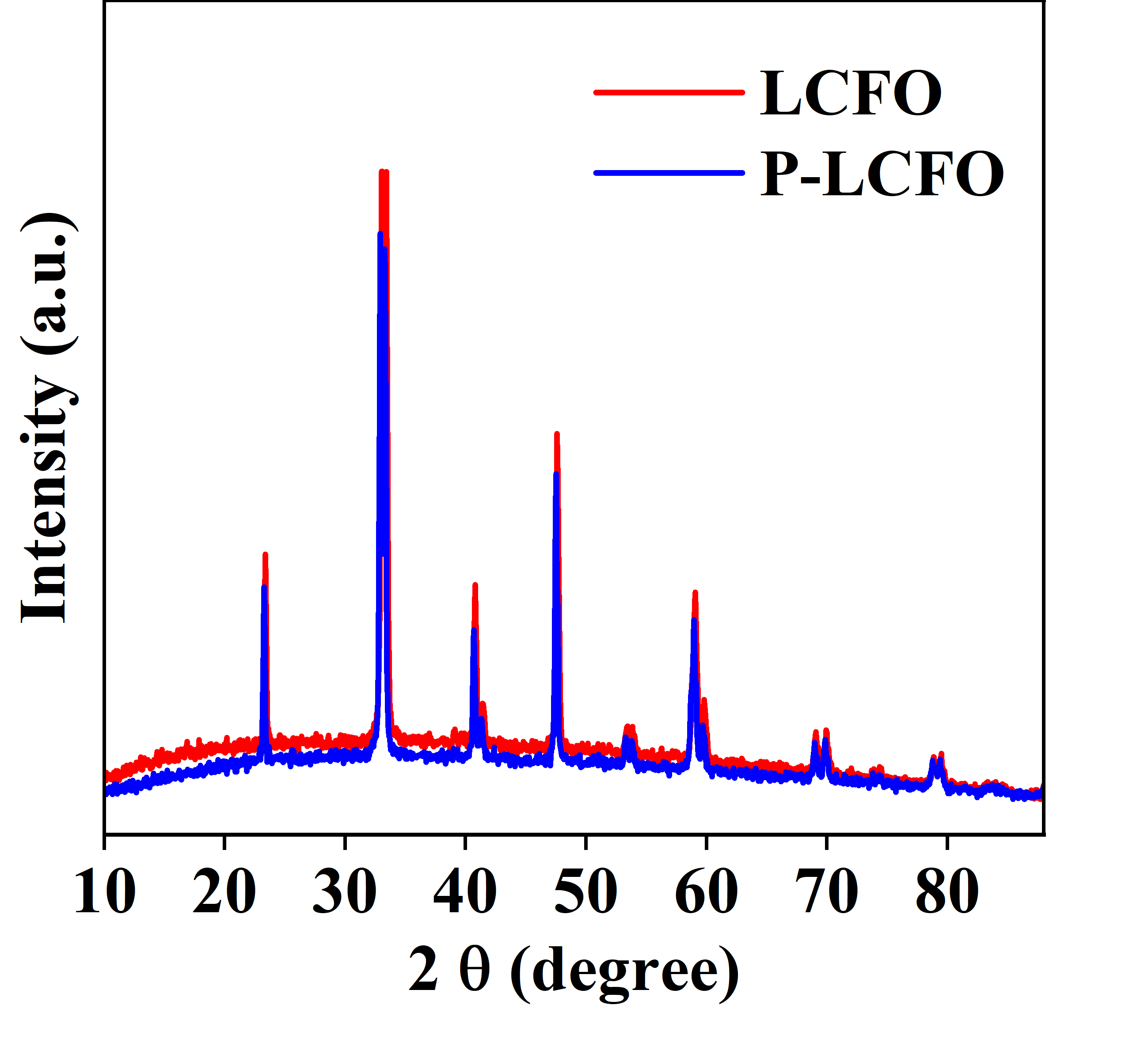


**Figure S2.** Comparison of XRD diffraction intensity between LCFO and P-LCFO.


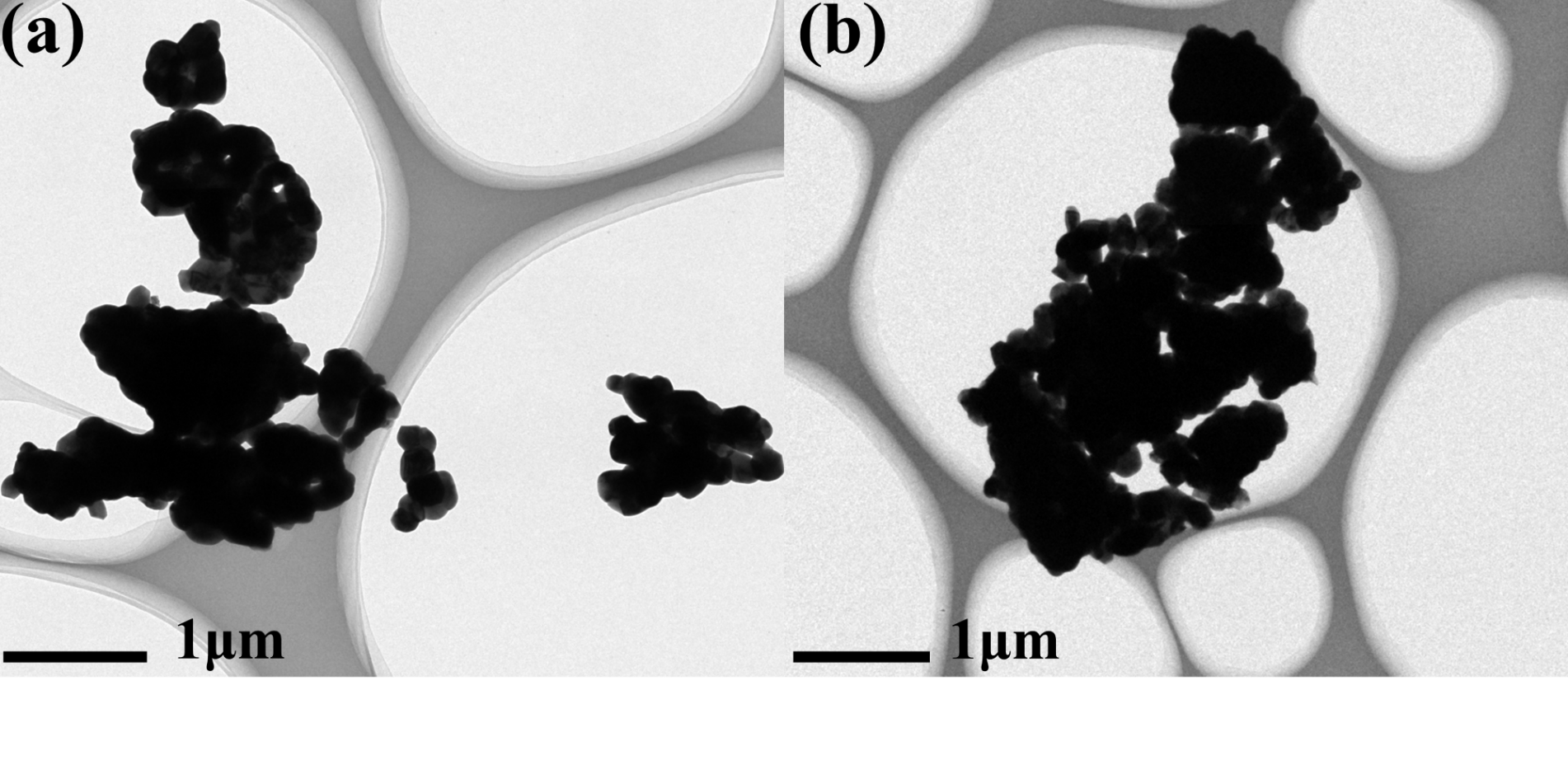


**Figure S3.** TEM images of (a) LCFO and (b) P-LCFO.


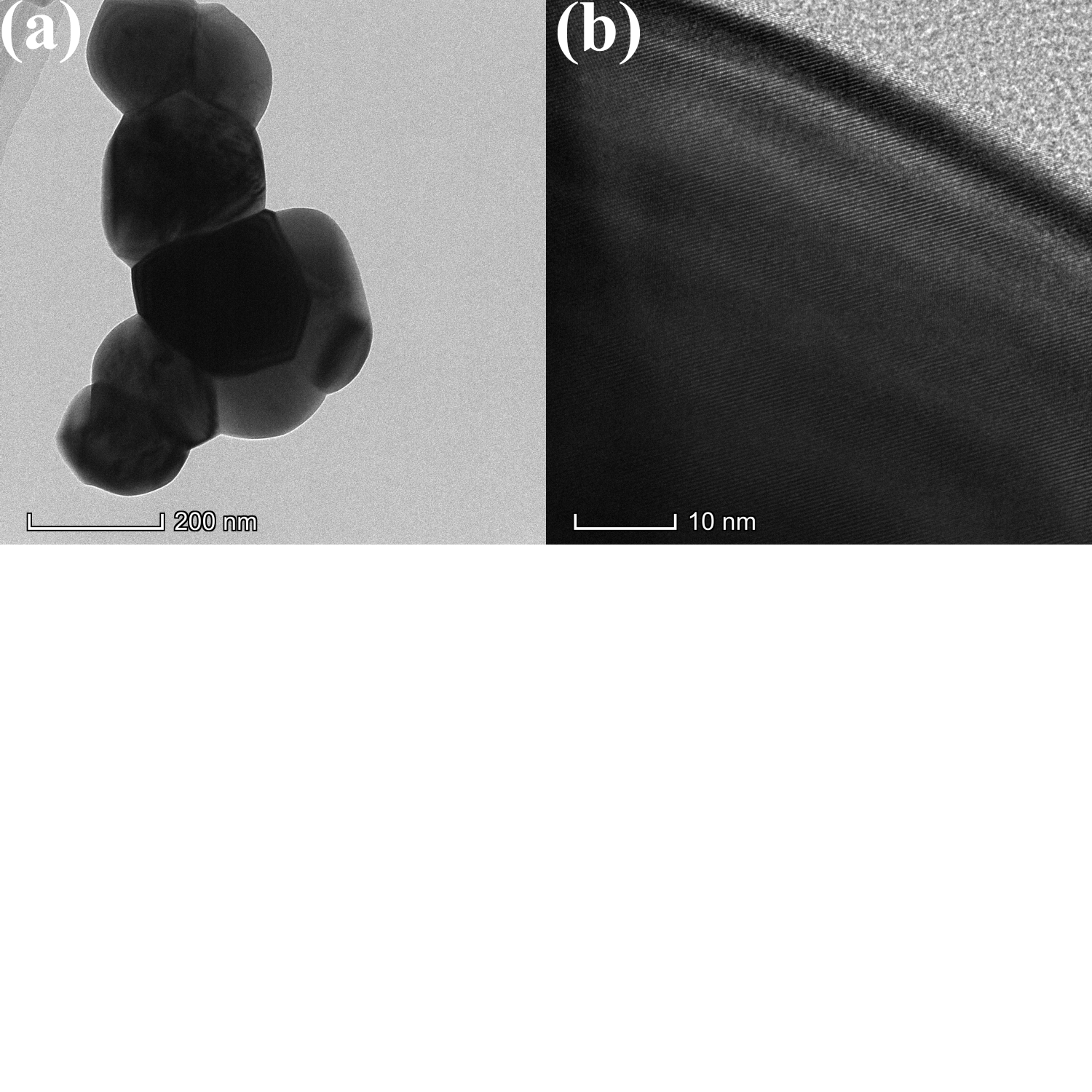


**Figure S4.** (a) TEM and (b) HRTEM images of LCFO.


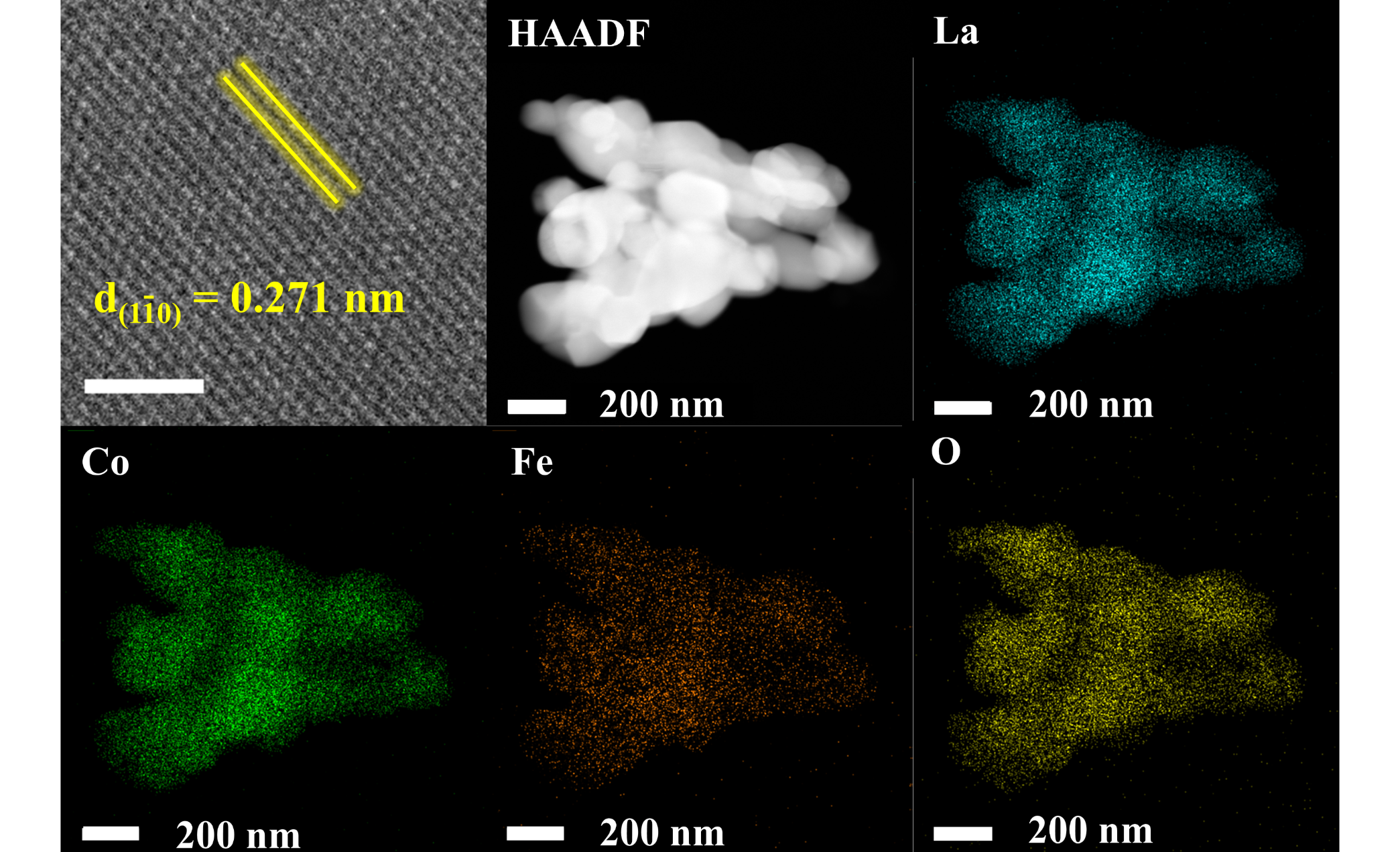


**Figure S5.** HRTEM, HAADF and EDS images of LCFO.


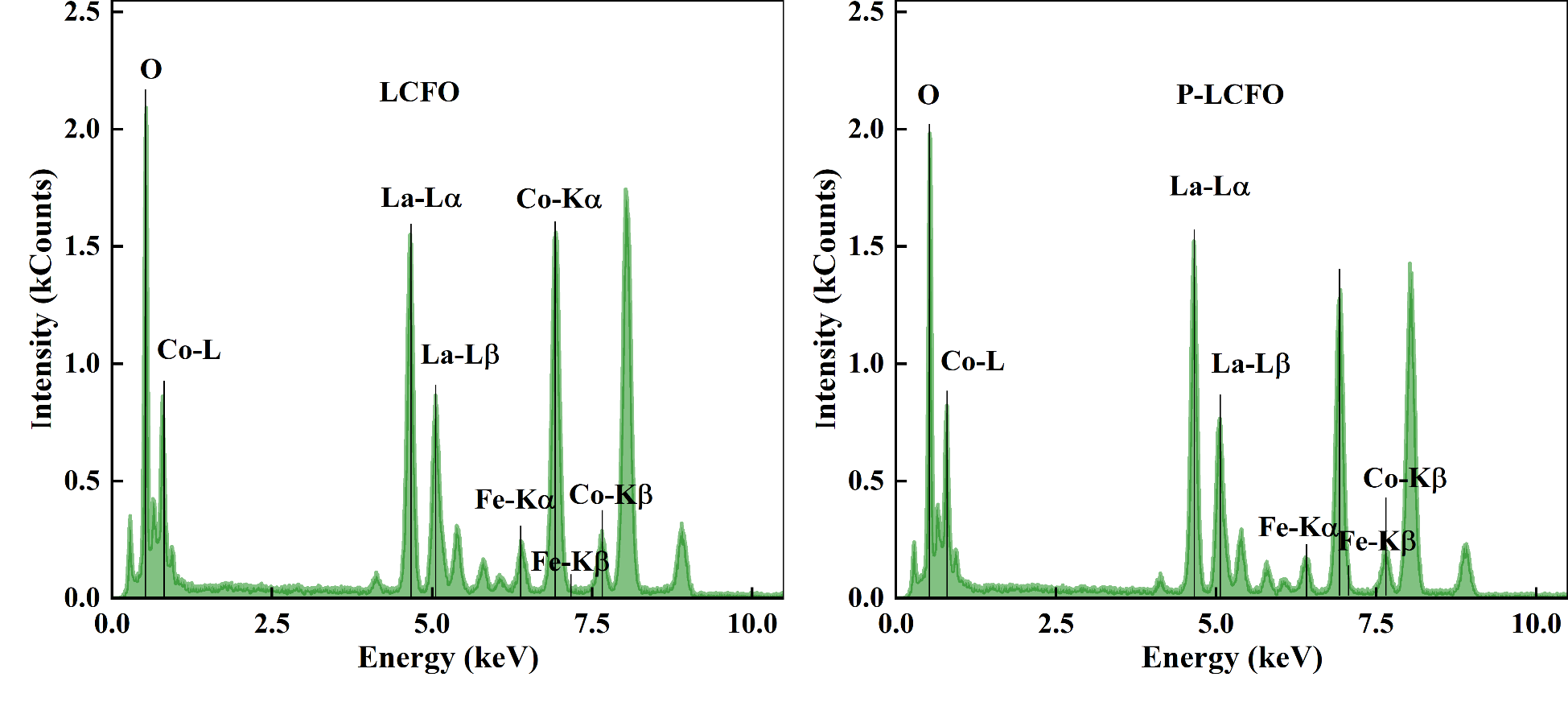


**Figure S6.** EDS patterns of LCFO and P-LCFO.


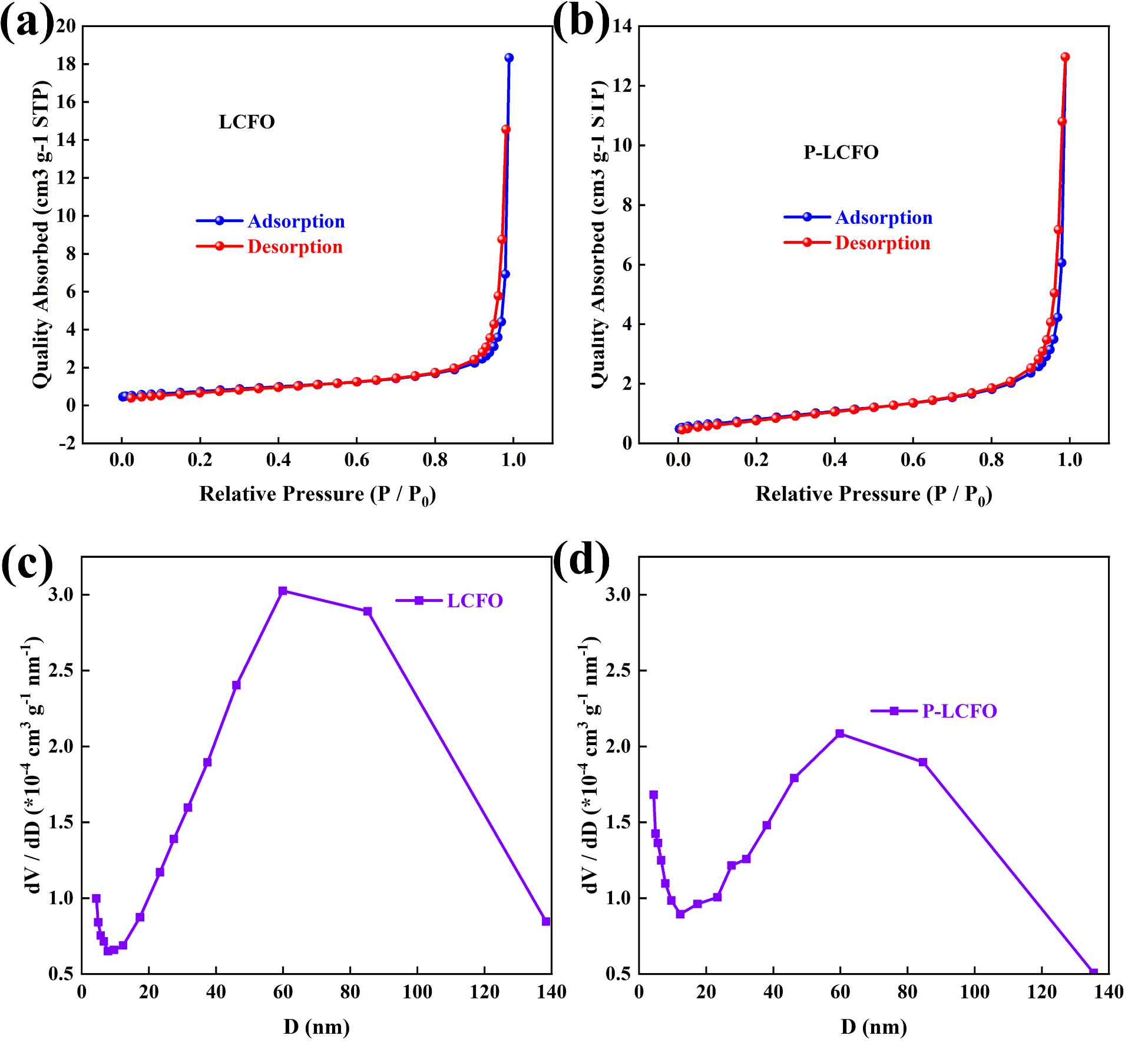


**Figure S7.** (a) and (b) N_2_ adsorption-desorption isotherms of LCFO and P-LCFO. (c) and (d) BJH pore size distribution curves of LCFO and P-LCFO.


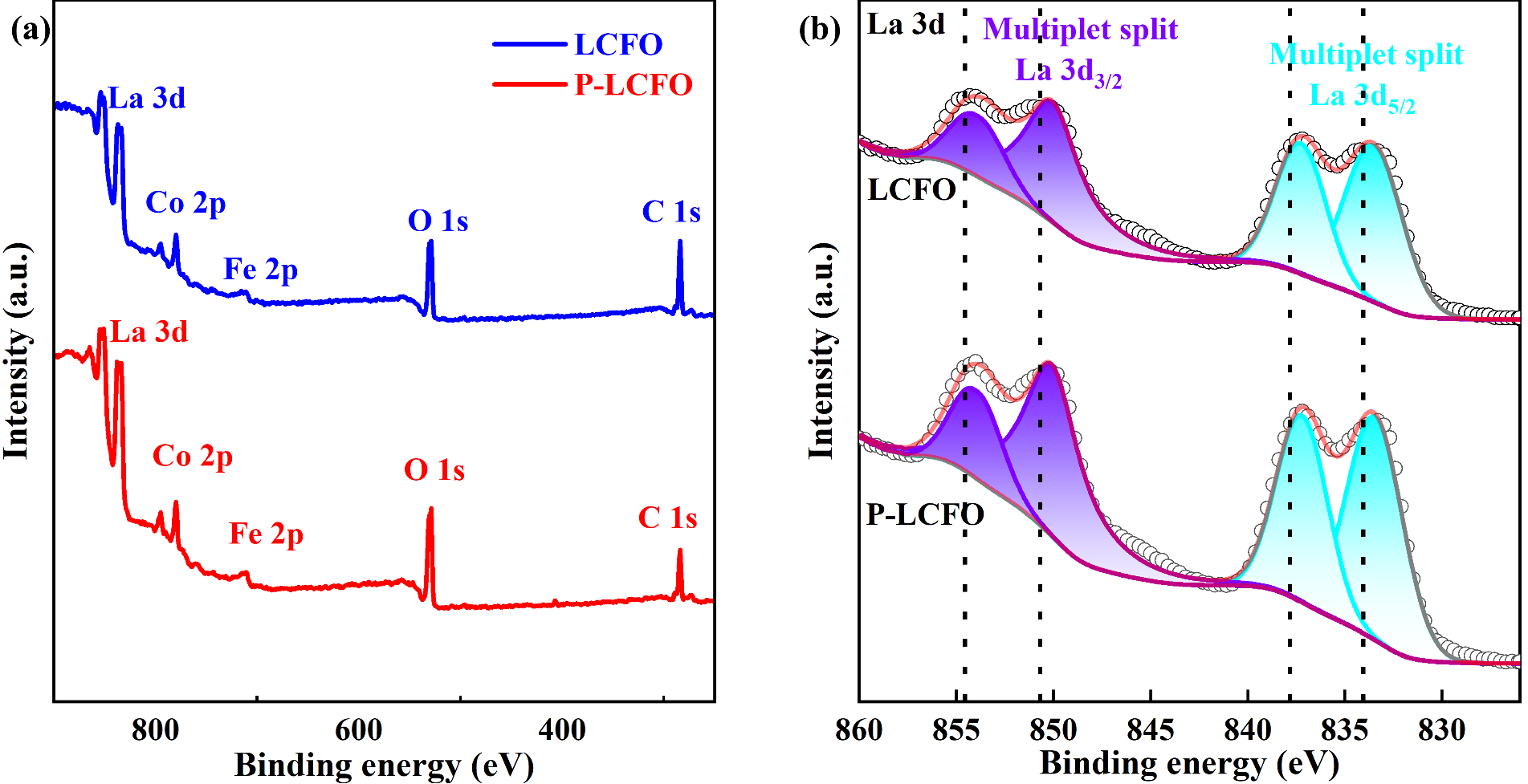


**Figure S8.** (a) XPS survey spectrum of LCFO and P-LCFO. (b) La 3*d* XPS spectra of LCFO and P-LCFO.


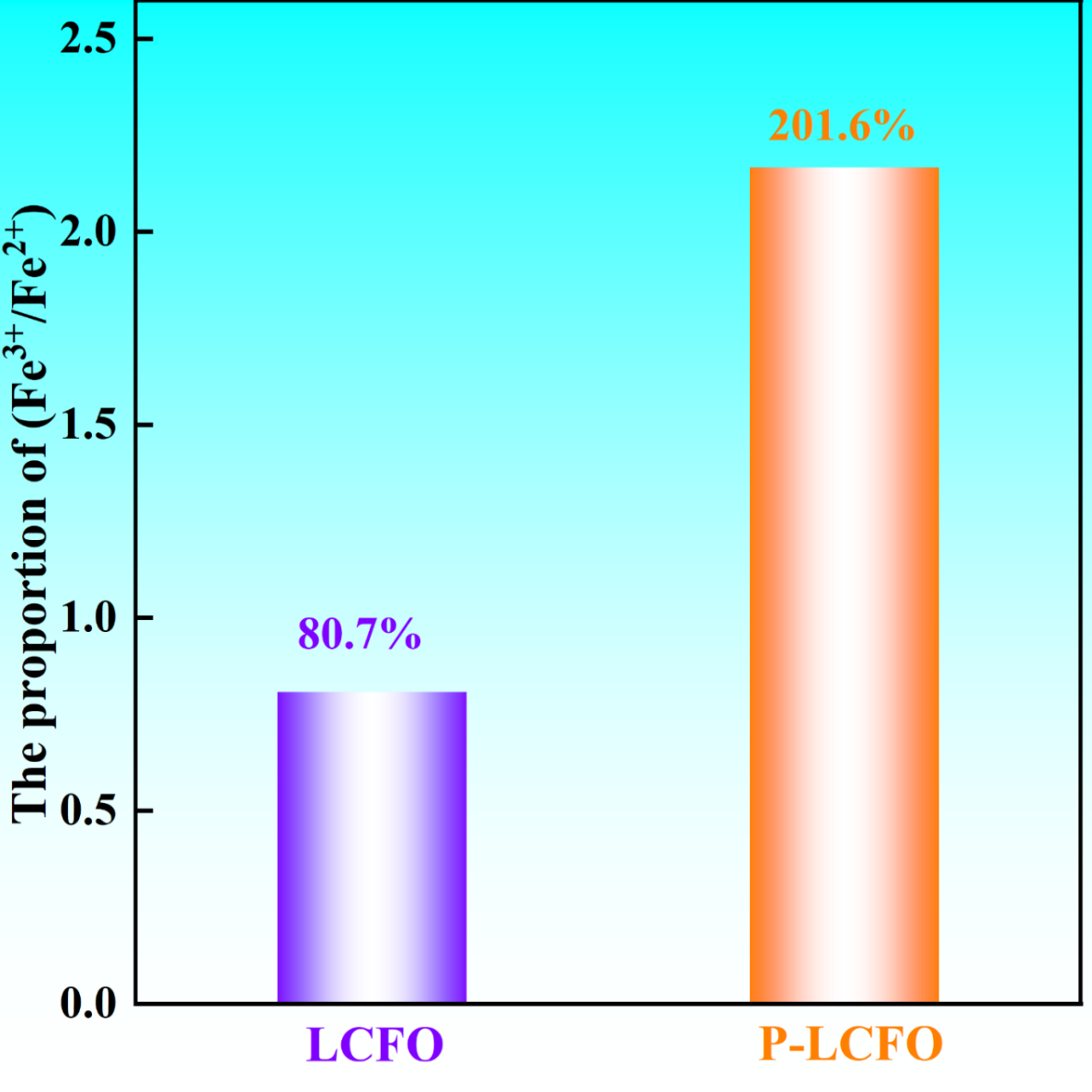


**Figure S9.** The proportion of Fe^3+^ /Fe^2+^ of LCFO and P-LCFO.


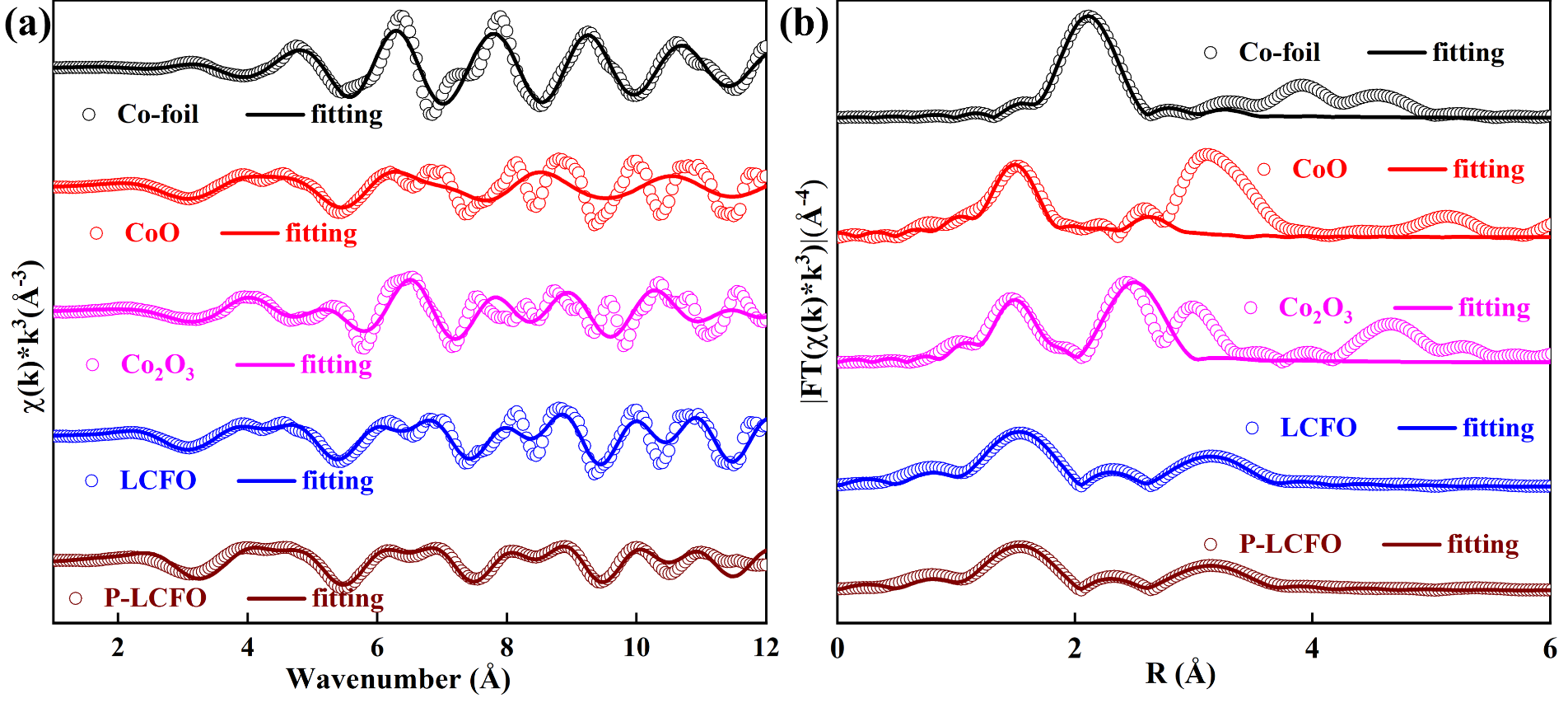


**Figure S10.** (a) Co K-edge fitting curves in K spaces. (b) Co K-edge fitting curves in R spaces.





**Figure S11.** Comparison of OER activity of perovskite oxides. Data are from **Table S2**.

**Figure S12.** An equivalent circuit diagram of EIS fitting.


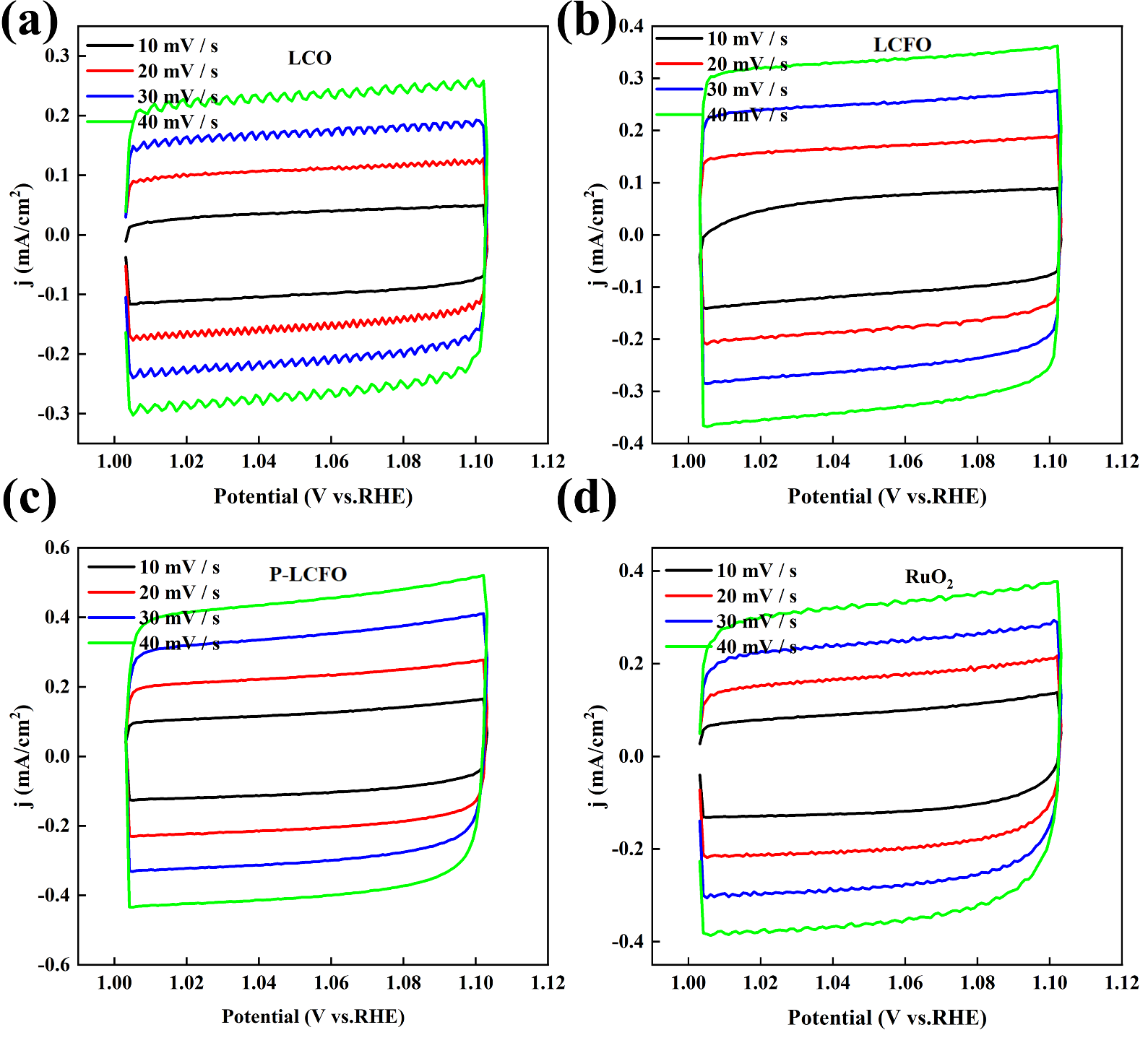


**Figure S13.** CV curves at different scan rates of (a) LCO, (b) LCFO, (c) P-LCFO, and (d) RuO_2_.

**
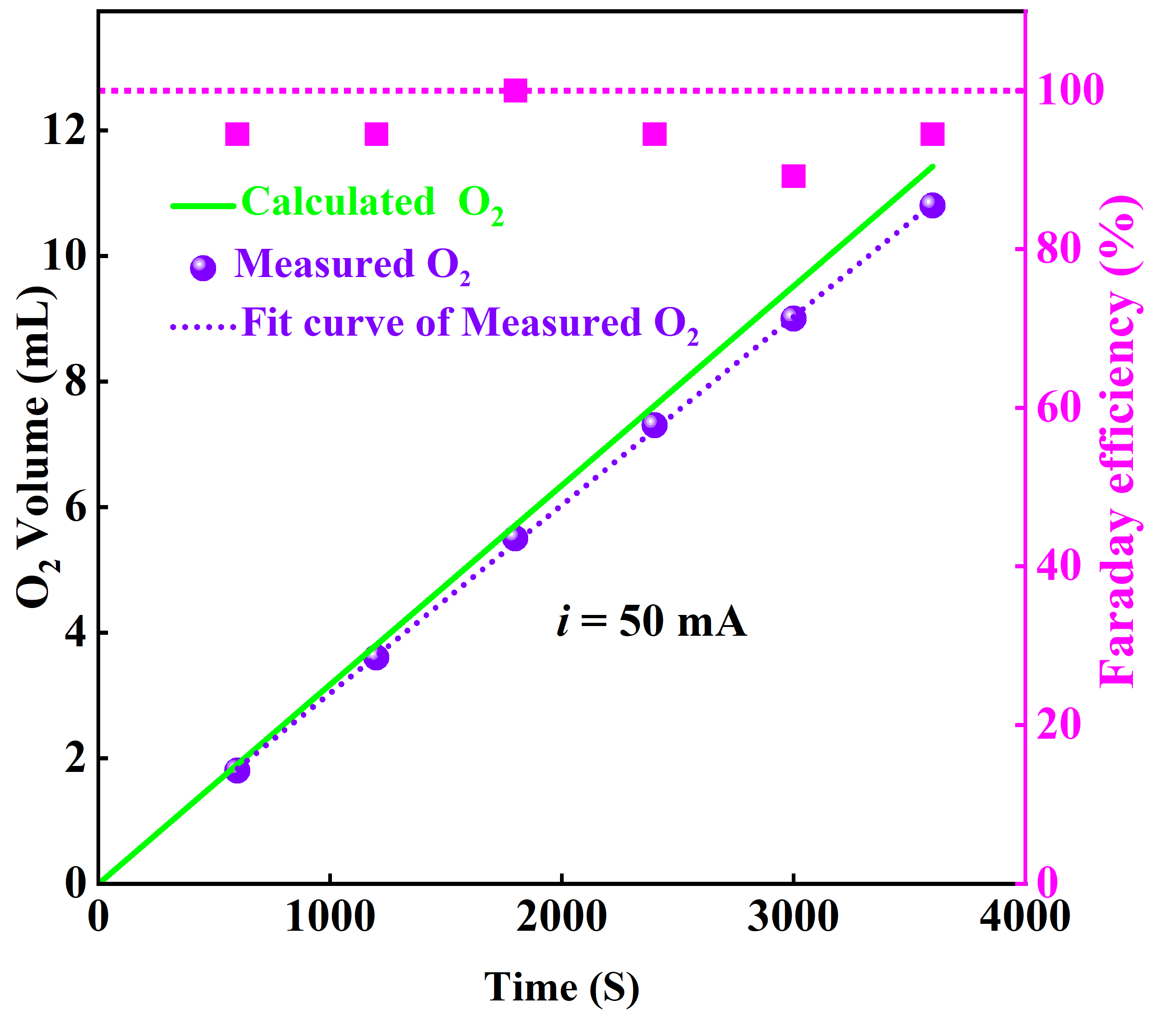
**

**Figure S14**. OER Faradaic efficiency measurement of P-LCFO at a current of 50 mA.


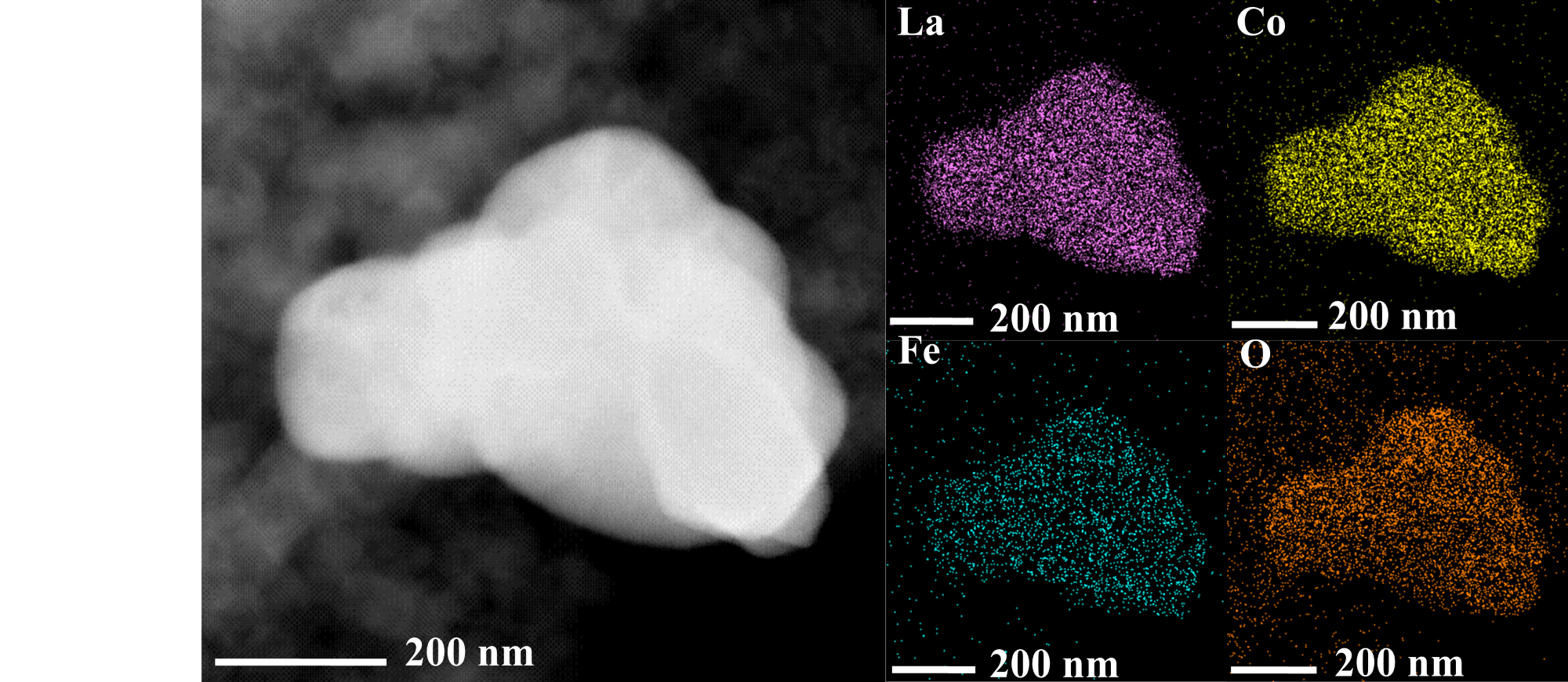


**Figure S15.** HAADF image and elemental mapping images of P-LCFO after the stability test.


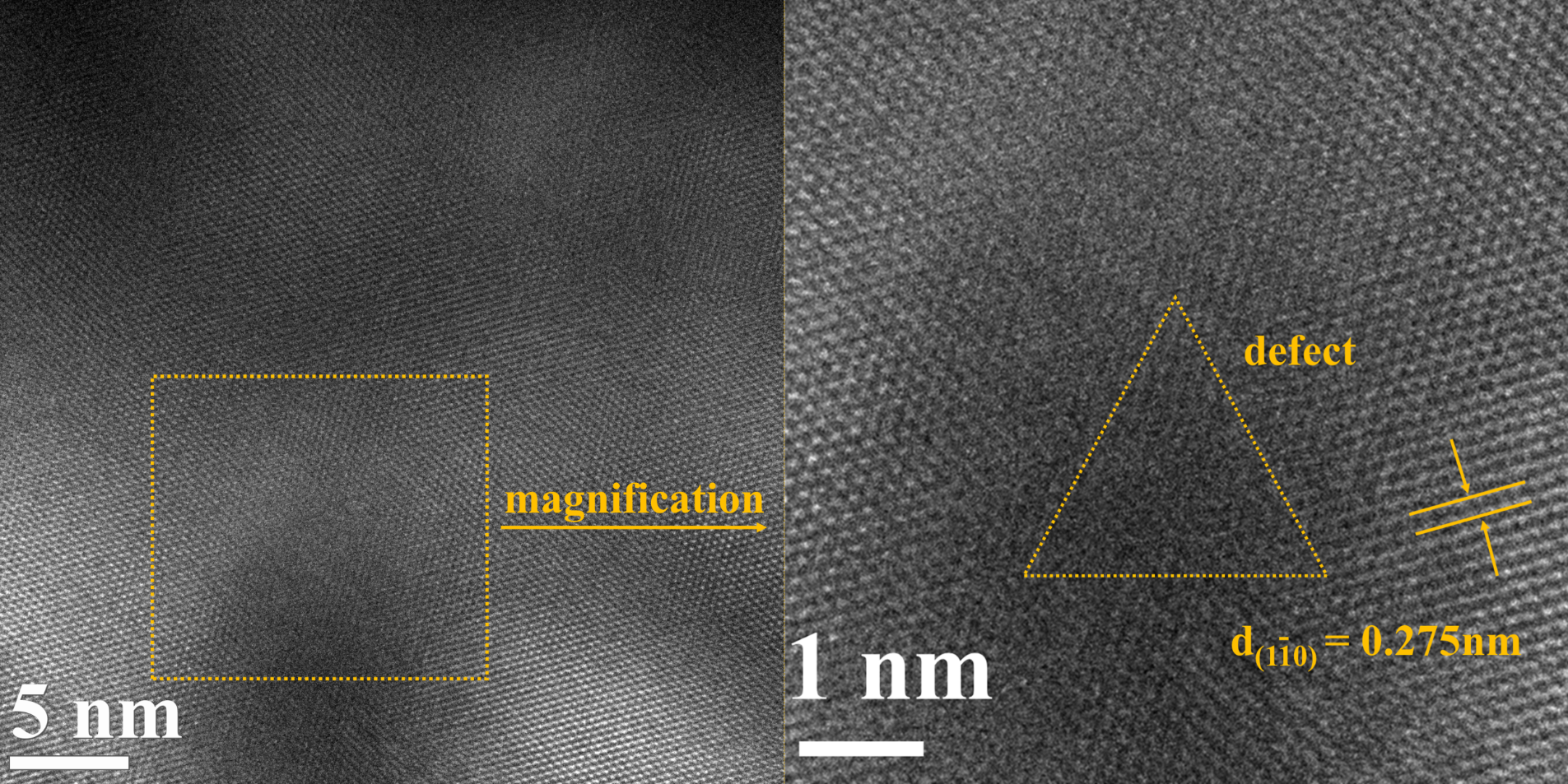


**Figure S16.** HRTEM images of P-LCFO after the stability test.


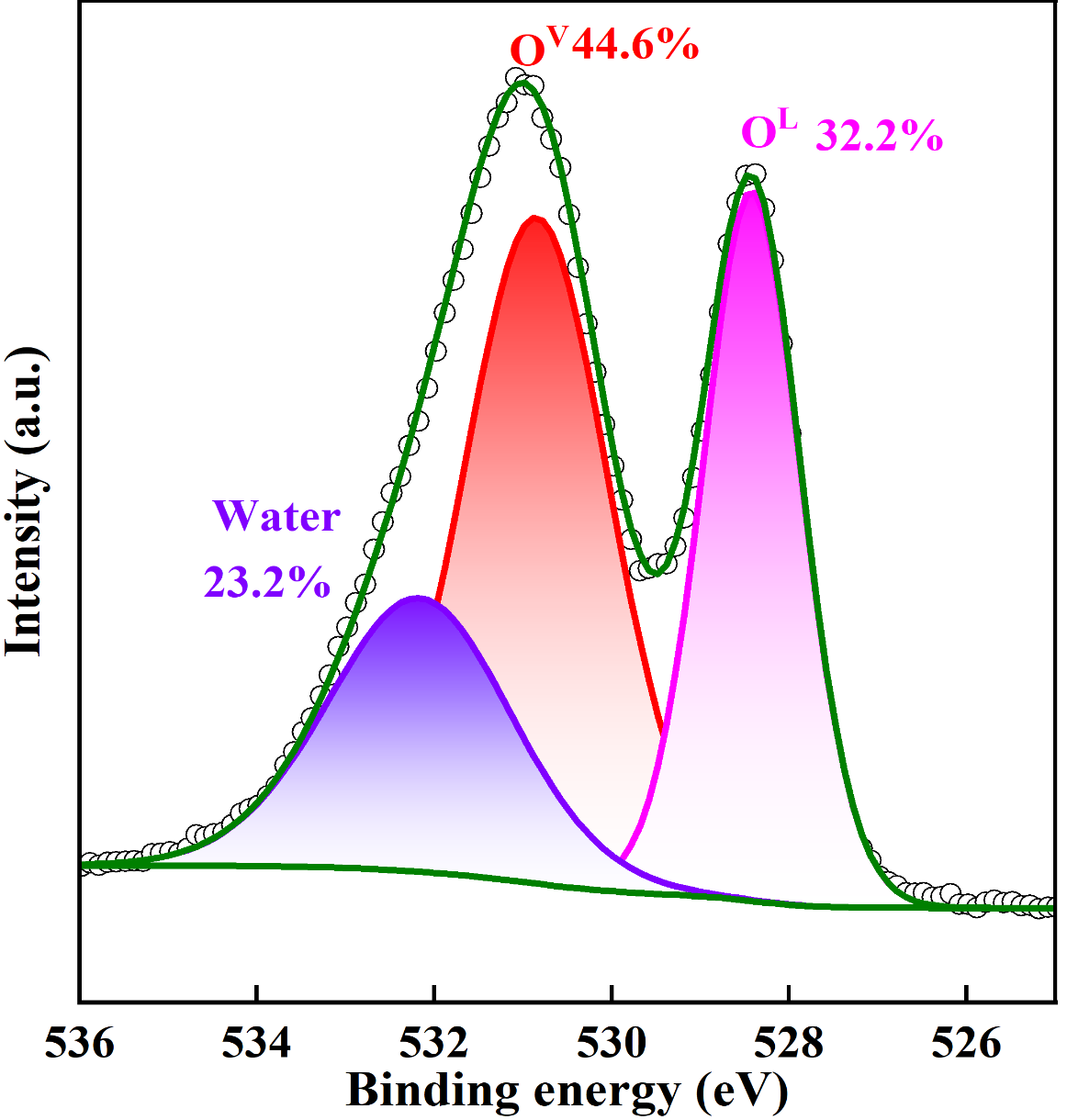


**Figure S17.** The O *1s* XPS spectrum of P-LCFO after stability testing

**Table S1.** EXAFS fitting parameters at the Co K-edge for LCFO and P-LCFO.

| **Sample**^a^ | **Path** | ***R***^b^ **(Å)** | ***N***^c^ | ***σ^2^*(10^3^Å^2^)**^d^ | ***ΔE_0_* (eV)**^e^ | **R factor** |
| --- | --- | --- | --- | --- | --- | --- |
| Co-foil | Co-Co1 | 2.49±0.013 | 12.00 | 0.0062 | -6.167 | 0.001 |
|  | Co-Co2 | 3.49±0.045 | 6.0 | 0.014 | -6.167 |  |
| CoO | Co-O | 2.14±0.012 | 5.4±0.4 | 0.0020 | -4.26 | 0.019 |
|  | Co-Co | 3.01±0.002 | 10.8±0.8 | 0.0021 | -4.26 |  |
| Co_2_O_3_ | Co-O | 1.93±0.040 | 4.8±0.6 | 0.0021 | -4.27 | 0.014 |
|  | Co-Co | 2.89±0.130 | 9.3±2.3 | 0.0069 | -4.27 |  |
| LCFO | Co-O | 1.92±0.003 | 5.5±0.4 | 0.002 | -2.83 | 0.010 |
|  | Co-La/Co | 3.31±0.007 | 2.8±0.5 | 0.006 | -2.83 |  |
| P-LCFO | Co-O | 1.95±0.021 | 4.8±0.9 | 0.0005 | 2.265 | 0.018 |
|  | Co-La/Co | 3.33±0.081 | 1.8±0.4 | 0.0008 | 2.265 |  |

^a^ **Ѕ_0_^2^** was fixed as 0.70. A reasonable range of EXAFS fitting parameters: 0.600 < **Ѕ_0_^2^** < 1.000; ***N*** > 0; *σ^2^* > 0 Å^2^ ; |***ΔE_0_***| < 15 eV; **R factor** < 0.02. ^b^***R***, distance between the absorber and backscatter atoms; ^c^***N***, coordination number; ^d^***σ^2^***, Debye–Waller factor to account for thermal and structural disorders; ^e^***ΔE_0_***, inner potential correction.

**Table S2.** Overpotential of perovskite oxides

| **Catlyst** | **Overpotential**  **(mV at 10 mA cm^-2^)** | | **References** |
| --- | --- | --- | --- |
| P-LCFO | **294** | This work | |
| LaCoO_3_ | 420 | *Adv. Mater. Interfaces* **2019***, 6* (1), 1801317. | |
| LaFeO_3_ | 510 | *Chem. Mater*. **2016**, *28* (6), 1691-1697. | |
| LaNiO_3_ | 435 | *Adv.Mater. Interfaces* **2019**, *6* (1), 1801317. | |
| LaMn_7_O_12_ | 300 | *Adv. Mater*. **2017**, *29* (4), 1603004. | |
| Sr_2_Fe_2_O_6_ | 480 | *Angew. Chem., Int. Ed*. **2019**, *58* (7), 2060-2063. | |
| La_0.2_Sr_0.8_FeO_3−X_ | 370 | *ACS Appl. Mater*. *Interfaces* **2018**, *10*, 11715. | |
| Ba_0.5_Sr_0.5_Co_0.8_Fe_0.2_O_3_ | 360 | *Inorg. Chem. Front*. **2020**, *7* (22), 4488– 4497. | |
| La_0.5_Sr_0.5_Ni_0.4_Fe_0.6_O_3−X_ | 330 | *Electrochim. Acta* **2017**, *246*, 997. | |
| Sr_2_Fe_0.8_Co_0.2_Mo_0.6_Co_0.4_O_6−X_ | 345 | *ChemSusChem* **2019**, *12*, 5111. | |
| BaCo_0.7_Fe_0.2_Sn_0.1_O_3−X_ | 450 | *Adv. Sci*. **2016**, *3*, 1500187. | |

**Table S3.** EIS fitting data for samples.

| Catalysts | R_s_ (Ω) | R_1_ (Ω) | R_ct_ (Ω) |
| --- | --- | --- | --- |
| LCO | 1.38 | 5.49 | 162.11 |
| LCFO | 1.58 | 5.66 | 77.53 |
| P-LCFO | 2.23 | 8.42 | 24.46 |
| RuO_2_ | 1.23 | 2.62 | 66.61 |
